# The impact of chronic rhinosinusitis on COVID-19 risk and outcomes: A systematic review and meta-analysis

**DOI:** 10.3389/fimmu.2023.1139031

**Published:** 2023-03-29

**Authors:** Abdullah Reda, Basant Ismail Lashin, Mustafa Mohammad Alaaraj, Moustafa Abouelkheir, Mahmoud Ibrahim Ahmed, Jaffer Shah, Amr Ehab El-Qushayri

**Affiliations:** ^1^Faculty of Medicine, Al-Azhar University, Cairo, Egypt; ^2^Faculty of Medicine, Banha University, Banha, Egypt; ^3^Faculty of Medicine, Alexandria University, Alexandria, Egypt; ^4^Trust Grade Foundation Doctor, Pilgrim Hospital, General Medicine, Boston, United Kingdom; ^5^Emergency Medicine Department, Pilgrim Hospital, United Lincolnshire NHS Trust, Lincolnshire, United Kingdom; ^6^Critical Care Clinical Fellow in Newcastle Upon Tyne Hospitals NHS Foundation Trust, Newcastle, United Kingdom; ^7^Medical Research Center, Kateb University, Kabul, Afghanistan; ^8^Faculty of Medicine, Minia University, Minia, Egypt

**Keywords:** chronic rhinosinusitis, steroids, COVID-19, coronavirus, prevalence, epidemiology

## Abstract

**Background:**

The impact of chronic rhinosinusitis (CRS) and subsequent steroid therapy on acquiring COVID-19 and severe outcomes remains controversial. Therefore, we conducted this systematic review and meta-analysis to provide cumulative evidence regarding the risk of COVID-19 and the impact of steroid therapy, length of hospital stay, mechanical ventilation, and mortality among CRC patients.

**Methods:**

We conducted a comprehensive electronic search strategy using the relevant keywords. The outcomes and risk factors of COVID-19 in CRS patients was estimated and compared to a healthy control group when applicable.

**Results:**

A total of seven studies were included, with an estimated prevalence of 6.5% (95% confidence interval (CI): 2.5-15.7) for COVID-19 in the CRS group. COVID-19 prevalence did not differ between CRS and controls (odds ratio (OR): 0.92; 95%CI: 0.84-1.01; p = 0.08). Moreover, using steroid/immunosuppressive therapy did not significantly increase the risk of acquiring COVID-19 in CRS patients compared to the control group (OR: 3.31; 95%CI: 0.72-15.26; p = 0.12). Length of hospital stay, mechanical ventilation, and mortality rates were comparable between the two groups. Furthermore, we found that male sex, cardiovascular morbidity, renal diseases, and hypertension were inversely associated with COVID-19 infection (p < 0.01).

**Conclusion:**

CRS had a neutral effect on acquiring COVID-19 and developing severe outcomes. However, further studies are needed.

## 1 Introduction

Chronic rhinosinusitis (CRS) is an inflammatory condition affecting the nasal cavity and related sinuses. Affected patients are characterized by various symptoms, including nasal discharge with and without facial pain, stuffy nose, and sense of smell-related conditions. Epidemiological data indicate the high prevalence of CRS in the general population, up to 12.5% (1). Evidence shows that nasal cavity-related pathological conditions can significantly lead to serious lower respiratory diseases, the main entry of different pathogens into the respiratory system (2, 3). Furthermore, established evidence shows that oral and nasal cavities are mainly responsible for introducing viral illnesses to the respiratory system, including the severe acute respiratory syndrome coronavirus 2 (SARS-CoV-2) (4). This might be due to specific antibody deficiency, exaggerated immune response, bacterial colonization, and epithelial barrier dysfunction (5, 6).

Since SARS-CoV-2 emerged and caused the coronavirus disease 2019 (COVID-19), different reports have indicated the global burden affection of the different aspects of the communities and their populations by the pandemic (7, 8). In addition, the disease has been associated with not only the development of acute respiratory distress syndromes but other conditions and complications that might even be life-threatening. Further, studies demonstrated that patients with underlying comorbidities are at risk of infection and developing severe COVID-19. These comorbidities include hypertension, diabetes mellitus (DM), obesity, and chronic obstructive pulmonary disease. Similarly, it has been shown that allergic rhinitis and asthma might be associated with an increased risk of COVID-19 and severe disease (9–11). This might be due to the influence of host immune functions on the infectivity and/or severity of COVID-19, based on the different affinities and expression levels of viral entry factors (12).

The exact role of CRS in COVID-19 is still unknown. Some studies were published to investigate this association. However, no cumulative evidence was found in these studies. For instance, some studies reported that CRS is not a significant risk factor for developing COVID-19 and severe disease. Moreover, it has been suggested that the condition might have a protective role against COVID-19 (12–16). On the other hand, other studies demonstrated that CRS increases the risk of hospitalization in COVID-19 patients (17, 18). Increased susceptibility to COVID-19 and increased risk of severe disease with CRS might be attributed to the high expression levels of viral entry genes, epithelial barrier dysfunction, and induced upper airway inflammatory condition. The main aim of this study is to provide cumulative evidence regarding the impact of CRS on COVID-19 infection and outcomes.

## 2 Methods

### 2.1 Search strategy

The steps of this systematic review were conducted according to the Preferred Reporting Items for Systematic reviews and Meta-Analyses (PRISMA) statement (19). We performed a comprehensive search of five electronic databases: PubMed, Scopus, ISI, google scholar, and VHL, for relevant studies published in the literature until July 10, 2022. We conducted another search on January 1, 2023 to check whether further studies were published. The search keywords and corresponding Medical Subject Headings (Mesh) were as follows (“COVID-19” OR “COVID 19” OR “novel coronavirus” OR “SARS-CoV-2”) AND (“chronic sinusitis” OR “chronic rhinosinusitis”). Moreover, we followed a manual search strategy as members looked up the references of relevant studies and reviews to find any relevant articles that might have been missed during the electronic search strategy.

### 2.2 Definition of endpoints

The current systematic review aims to identify the impact of CRS on acquiring COVID-19 and related severe outcomes of previously affected patients. Accordingly, we will mainly assess the prevalence of COVID-19 in CRS patients and compare it to the control group of non-CRS individuals. The intervention group will include patients with CRS, while the control one will include healthy individuals with no CRS or another control group. We will also estimate the rates of hospitalization, intensive care unit (ICU) admissions, mechanical ventilation, and mortality, together with the length of hospital stay. We will also assess the impact of steroids/immunosuppressive therapy used by CRS patients on acquiring COVID-19. These outcomes will be compared to the control group whenever possible.

Other secondary endpoints of the current study will include the risk factors for developing COVID-19 and the difference in symptomatology and COVID-19 positive and negative patients with CRS.

### 2.3 Inclusion and exclusion criteria

We drafted our criteria based on our intended outcomes. Accordingly, we included articles that 1) were original, including cross-sectional and cohort studies 2) included human populations and 3) investigated the prevalence of COVID-19 (PCR-confirmed) or any related outcome in CRS patients or compared the prevalence between CRS and non-CRS (control) individuals. On the other hand, we excluded articles that 1) were not original (like different types of reviews, abstract-only articles, editorials, thesis, protocols, and conference papers), or 2) included limited sample populations, like case reports and case series, 3) were not human studies (like *in vivo* and *in vitro* studies), 4) did not include CRS patients, 5) did not report any COVID-19 related outcomes among these patients, or 6) reported other outcomes related to laboratory data or genetic information.

### 2.4 Screening process

After we finished the search strategy, all citations from all databases were grouped into a single Endnote library to help us remove all potential duplicates among these databases. Following this step, we used an Excel sheet and gave each citation an ID to help identify and facilitate the screening of the different citations. The screening process was divided into two main steps, including title/abstract followed by full-text screening. Both of these steps were conducted by at least two study members. To prevent bias in selection, each member’s screening results were blinded from the other. However, after they were finished, the senior member grouped and compared the results of each member and highlighted the differences to be discussed to reach a final decision about whether to include or exclude the article according to the inclusion and exclusion criteria.

### 2.5 Data extraction and quality assessment

A pilot extraction sheet was drafted based on the intended outcomes. It was then tested and modified whenever needed throughout the extraction process to enhance the quality of reporting and facilitate the extraction process. The sheet mainly consisted of three parts. The first part was made for the baseline characteristics (including study reference, study design, sample size, age, and gender (male %) of each group, and method of COVID-19 diagnosis. The second part was developed for the study outcomes, including the prevalence of COVID-19, hospitalization, length of hospital stay, mechanical ventilation, ICU admissions, and mortality rates in the CRS and control groups. This part also included the symptoms of each group and the risk factors between COVID-19 positive and negative CRS patients. The third part included the domains of the Newcastle–Ottawa scale (NOS) for assessing the quality of non-randomized studies (Cohorts and Case-controls). The tool mainly assessed the quality of each included study by three domains, including selection, compatibility, and outcomes, which were divided into eight items. The highest score for any study was nine, and studies were stratified based on their totally estimated scores as follows: 1) 0-3 very high risk of bias, 2) 4-6 moderate risk (fair quality), and 3) 7-9 high quality. We also used the modified the NOS for cross-sectional studies, which was also composed of three main domains, divided into seven items with a maximum score of ten. The extraction process was also conducted in a blinded approach to reduce the risk of bias in extraction. A discussion was also made whenever there was a disagreement to reach a final decision.

### 2.6 Statistical analysis

Data analysis was conducted using the Comprehensive Meta-analysis (CMA, Version 3) software. Random or fixed models were chosen based on the heterogeneity of included studies. We identified heterogeneity as P-value < 0.1, or I (2) ≥ 50%. We expressed data in rates (%) for data presented in events/totals and measuring the prevalence of outcomes. We also compared the outcomes between the two study groups, and data was represented by odds ratio (OR) and 95% confidence interval (CI). A P-value < 0.05 was considered statistically significant.

## 3 Results

### 3.1 Study selection

**Figure 1:**
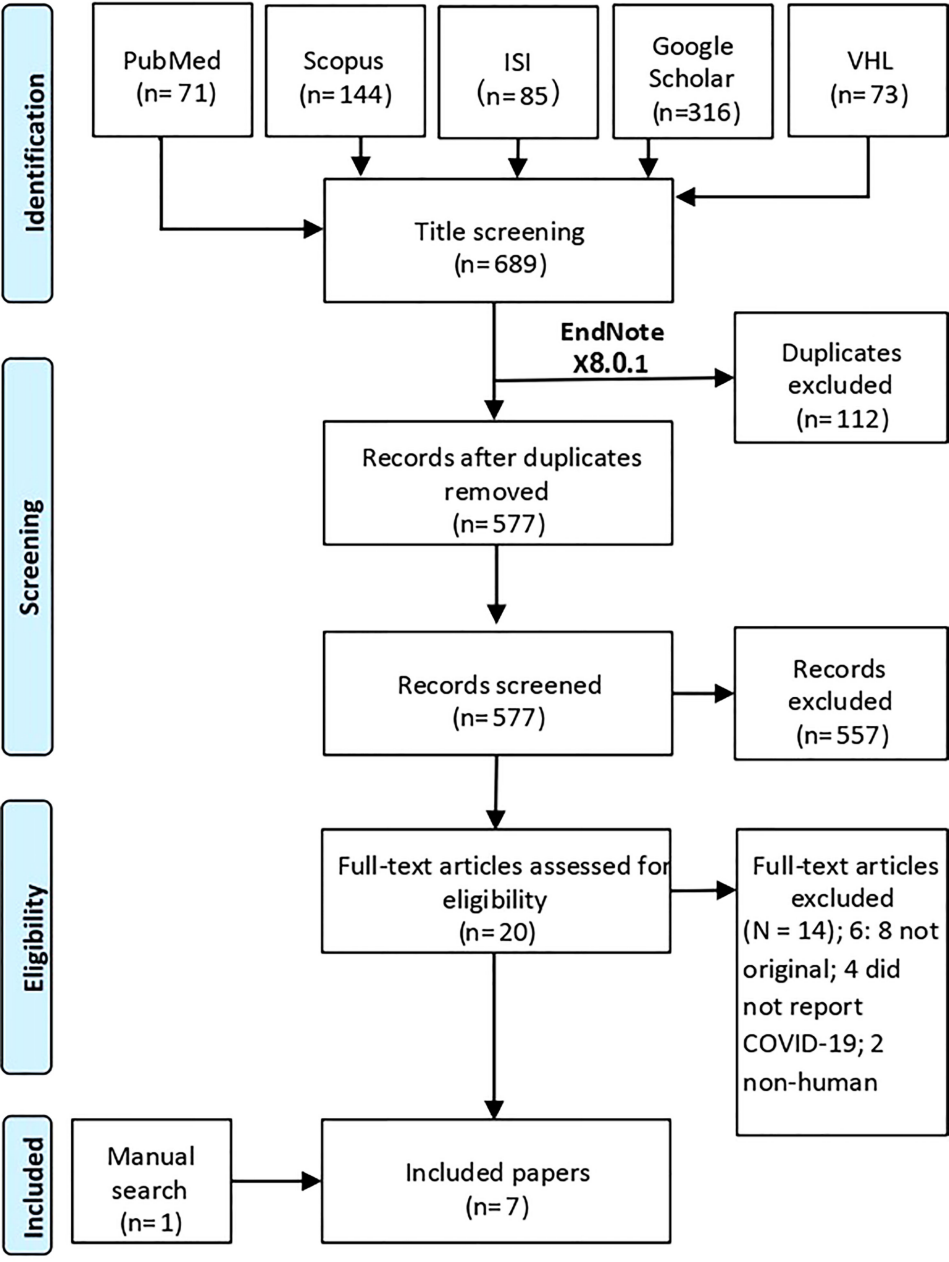
PRISMA flow diagram.

A total of 690 studies were imported for a title and abstract screening, and after removing duplicates, 578 studies were retained. These articles were screened by title/abstract screening, and as a result, only 21 studies were deemed eligible for full-text review. From the full-text review, seven papers met our criteria and were extracted. The rest of these articles were excluded because they were not original, did not include any of our reported outcomes, or was duplicated with another included article. We detailed this process in the PRISMA flow chart in **Figure 1**.

### 3.2 Study characteristics and quality assessment

A total of seven studies, including five cross-sectional (13, 15–17, 20) and two cohort studies (18, 21), were finally retrieved. The included studies’ sample size remarkably varied, ranging from 52 to 12323 in the CRS group and 942 to 207636 in the control group. Similarly, the age of included participants was remarkably variable among included studies. Besides, the proportion of women was comparable among all CRS and control groups. All COVID-19 patients were diagnosed by reverse transcription-polymerase chain reaction. All baseline characteristics are presented in **Table 1**.

**Table 1:** Baseline characteristics of included studies.

Reference	Study design	Sample size (N)		Mean (SD) age (years)		Male gender (%)		COVID-19 diagnosis
		CRS	Control	CRS	Control	CRS	Control	
**Akhlaghi et al.** (16)	Cross-sectional	207	–	15 – >50		57.5	–	PCR
**Faiq et al.** (20)	cross-sectional	150	–	10 – 70		47.3	–	PCR
**Sbeih et al.** (17)	cross-sectional	52	942	58.14 (2.27)	54.63 (0.62)	32.69	48.94	PCR
**Wang et al.** (13)	cross sectional	72	1100	60.5 (46.25 - 68)*	61 (48 - 68.75)*	59.7	48.5	PCR
**Workman et al. (****15****)**	cross-sectional	12282	12282	53	53.1	44.6	44.6	PCR
**Lee et al.** (18)	Cohort	12323	207636	55 (19.6)	49.1 (19.9)	47.8	47.7	PCR
**Miller et al. (****21****)**	Cohort	1707	–	55.4 (17.2)	–	39.4	–	PCR

CRS, Chronic rhinosinusitis; PCR, Polymerase chain reaction; *data presented in median (Interquartile range).

–, not reported.

Results of the quality assessment of each study and domains of the NOS tool are presented in **Supplementary Tables 1**, **2**.

### 3.3 COVID-19 prevalence

**Figure 2:**
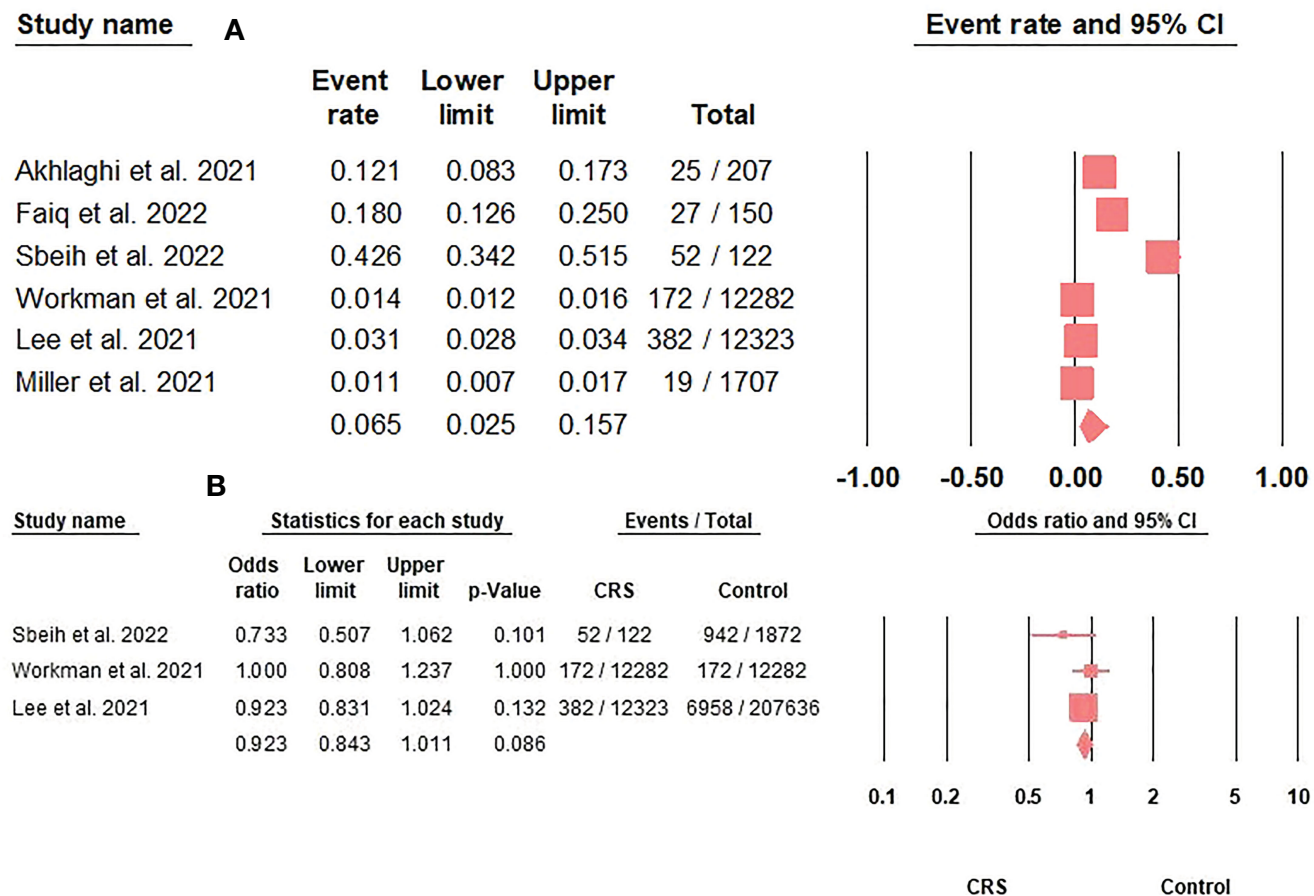
**(A)**. The COVID-19 prevalence rate in CRS patients represented by the event rate and 95% confidence interval. Figure 2 **(B)**. The association between COVID-19 prevalence and CRS represented by the odds ratio and 95% confidence interval.

The prevalence of COVID-19 in CRS patients was 6.5% (95%CI: 2.5-15.7) (**Figure 2A**). Furthermore, COVID-19 prevalence did not differ between CRS and controls (OR: 0.92; 95%CI: 0.84-1.01; p = 0.08) (**Figure 2B**).

### 3.4 COVID-19 outcomes

#### 3.4.1 COVID-19 hospitalization

Around one-fifth (20.8%; 95%CI: 3.7-84) of CRS patients with COVID-19 required hospitalization (**Table 2**). Moreover, COVID-19 hospitalization rates were significantly higher in CRS patients than in controls (38.46% versus 25.82%; p = 0.03) (17).

**Table 2:** Prevalence of outcomes and symptoms of COVID-19 patients with chronic rhinosinusitis.

Variables	Number of studies	Model	Prevalence (95% CI)
Outcomes			
**Hospitalization**	2	Random	20.8% (3.7-64)
**Mechanical ventilation**	2	Fixed	5.4% (2.5-11.6)
**ICU admission**	2	Fixed	6.7% (2.8-15)
Symptoms			
**Fever**	3	Random	67% (30-91)
**Cough**	3	Random	55% (32-75)
**Anosmia**	3	Random	49.5 (27-73)
**Nasal obstruction**	2	Fixed	44.4% (31.4-58.2)
**Fatigue**	3	Random	41.5% (21-65)
**Dyspnea**	3	Fixed	36% (29-44)
**Anorexia**	2	Fixed	19.6% (14-28)
**Diarrhea**	3	Fixed	16.7% (11-24)
**Nausea and vomiting**	3	Fixed	7% (4-13)

#### 3.4.2 Length of hospital stay in COVID-19

The length of hospital stay in COVID-19 patients with CRS (n= 72) was similar to the controls(n=1100) [Median: 18 (IQR: 15 – 23) versus 18 (12 – 21.5) days, respectively] (13).

#### 3.4.3 Mechanical ventilation in COVID-19

**Figure 3:**
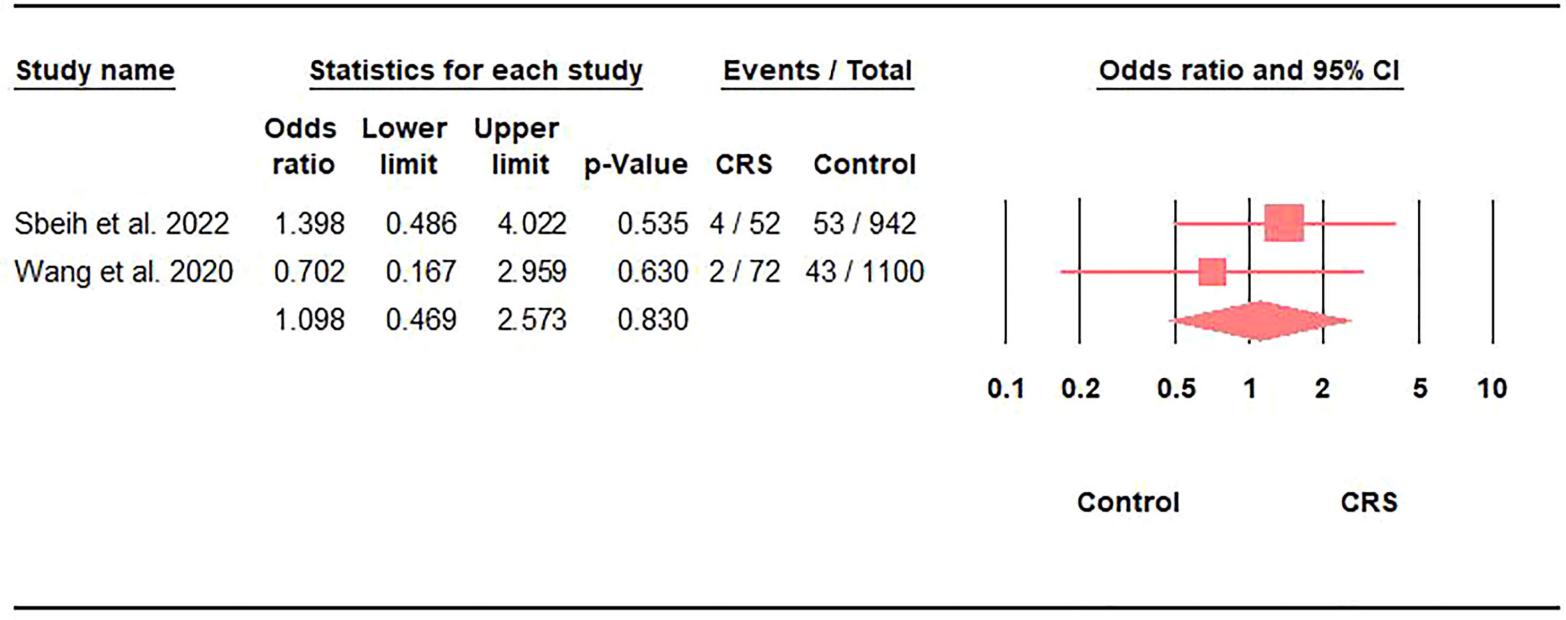
Association between the prevalence of mechanical ventilation in COVID-19 and CRS represented by the odds ratio and 95% confidence interval.

Only 5.4% (95%CI: 2.5-11.6) of CRS patients with COVID-19 needed mechanical ventilation (**Table 2**). However, comparing mechanical ventilation rates against controls did not reveal any significant differences (OR: 1.1; 95%CI: 0.47-2.57; p = 0.8) (**Figure 3**).

#### 3.4.4 ICU in COVID-19

The prevalence of CRS with COVID-19 infection admitted to the ICU was 6.7% (95%CI: 2.8-15) (**Table 2**).

#### 3.4.5 Mortality in COVID-19

COVID-19 mortality in CRS patients (n=52) was lower, but not statistically significant than controls (n=942) (2.9% vs. 4.6%, P-value > 0.05) (17).

### 3.5 Impact of steroid/immunosuppressive therapy

**Figure 4:**
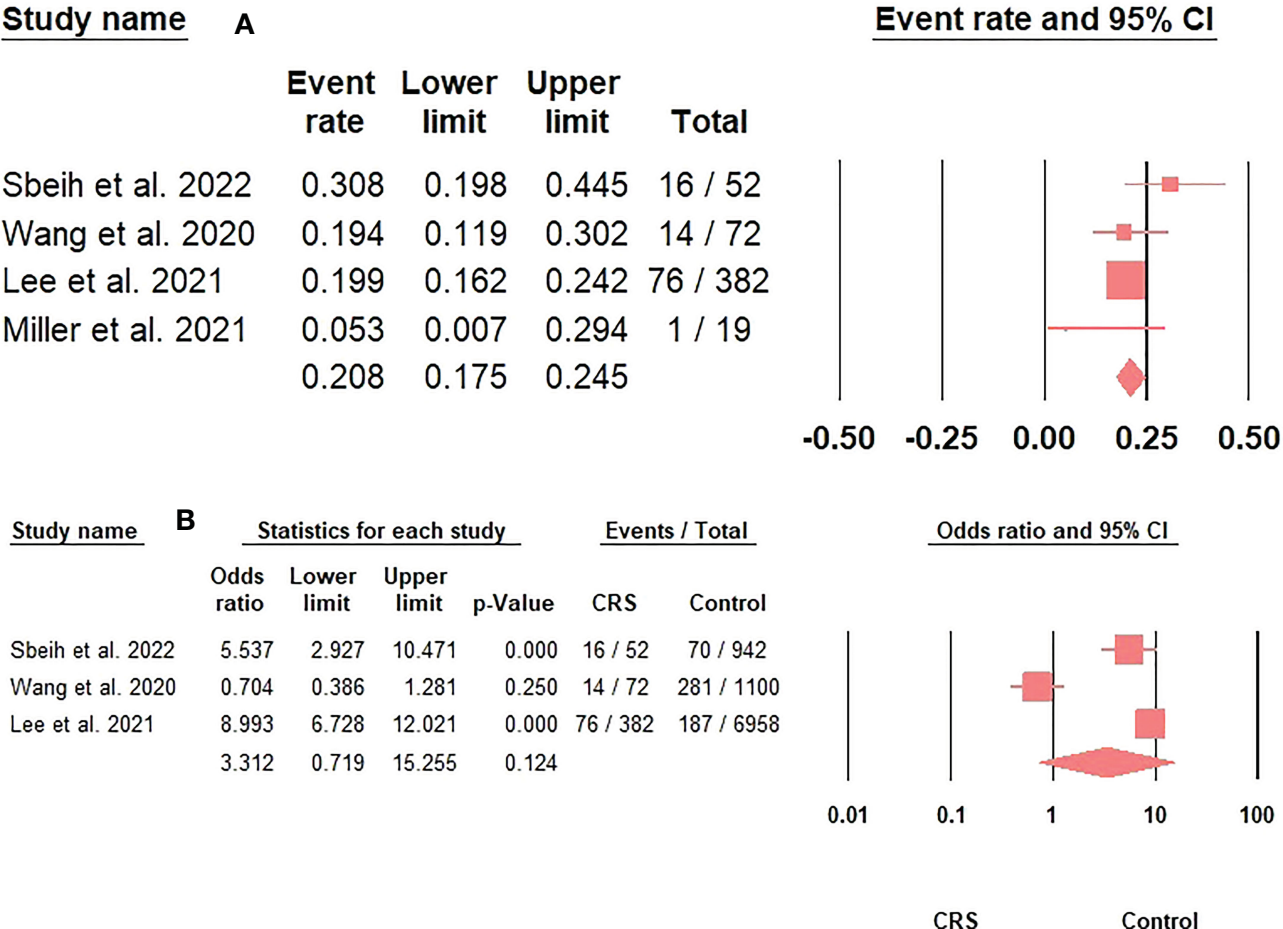
**(A)**. The prevalence of steroids/immunosuppressive therapy use in CRS patients with COVID-19 represented by the event rate and 95% confidence interval. Figure 4 **(B)**. The association between steroids/immunosuppressive therapy use in CRS patients and acquiring COVID-19 represented by the odds ratio and 95% confidence interval.

The prevalence of steroid/immunosuppressive therapy administration in CRS patients with COVID-19 was 20.8% (95%CI: 17.5-24.5) (**Figure 4A**). Moreover, using steroid/immunosuppressive therapy did not significantly increase the risk of acquiring COVID-19 in CRS patients compared to the control group (OR: 3.31; 95%CI: 0.72-15.26; p = 0.12) (**Figure 4B**). Finally, CRS patients treated with inhaled corticosteroids had a greater risk of developing severe COVID-19 outcomes than non-CRS patients (25.7% versus 13.3%; adjusted OR: 2.24; 95%CI: 1.16-4.20) (18).

### 3.6 COVID-19 risk factors in CRS patients

We found that male sex, cardiovascular morbidity, renal diseases, and hypertension were inversely associated with COVID-19 infection (p < 0.01). However, nasal polyps, cerebrovascular disorders, DM, and steroids/immunosuppressive therapy use were comparable between COVID-19 positive and negative patients (p > 0.05) (**Table 3**).

**Table 3:** Risk factors and symptoms of COVID-19 with chronic rhinosinusitis compared to the control group.

Variables	Number of studies	Model	OR (95% CI)	P value
Risk factors				
**Hypertension**	2	Fixed	0.57 (0.64-0.83)	<0.001
**Male sex**	2	Fixed	0.7 (0.58-0.86)	0.001
**Renal diseases**	2	Fixed	0.59 (0.41-0.85)	0.004
**Cardiovascular diseases**	2	Fixed	0.55 (0.43-0.72)	<0.001
**Cerebrovascular diseases**	2	Random	1.59 (0.23-11)	0.64
**Diabetes mellitus**	2	Fixed	0.91 (0.73-1.14)	0.41
**Steroid/immunosuppressive therapy**	2	Fixed	1.12 (0.87-1.44)	0.39
**Nasal polyp**	2	Random	1.55 (0.39-6.15)	0.53
Symptoms				
**Anorexia**	2	Fixed	0.88 (0.55-1.39)	0.58
**Cough**	2	Fixed	0.84 (0.57-1.22)	0.38
**Diarrhea**	2	Fixed	0.93 (0.58-1.5)	0.77
**Dyspnea**	2	Fixed	0.84 (0.58-1.23)	0.37
**Fatigue**	2	Fixed	1.05 (0.7-1.57)	0.8
**Fever**	2	Random	0.98 (0.26-3.71)	0.98
**Nausea and vomiting**	2	Fixed	0.9 (0.41-1.98)	0.8

### 3.7 COVID-19 symptoms

We found that fever, cough, and anosmia were the common COVID-19 symptoms in CRS COVID-19 patients 67%, 55%, and 49.5% (**Table 2**). On further comparison of COVID-19 symptoms between COVID-19 patients in the CRS and control population, we did not find any significant differences between the two groups regarding fever, fatigue, anorexia, cough, diarrhea, dyspnea, and nausea and vomiting (p > 0.05) (**Table 3**).

## 4 Discussion

The main aim of this study was to investigate the impact of CRS on susceptibility to COVID-19 and related severity. Our findings indicate that the rates of COVID-19 were comparable in the CRS and non-CRS groups, indicating that CRS does not impact the susceptibility to SARS-CoV-2 infection. Moreover, we found that the prevalence of COVID-19 cases among patients with CRS was 6.5%. This rate is not considered high, especially during times of the pandemic, which might furtherly indicates the neutral impact of CRS on the development of COVID-19. However, it is hard to compare prevalence rates because of different inclusion criteria and trends of COVID-19 per country.

CRS is characterized by type 2 inflammation and is associated with increased levels of interleukin (IL)-4, IL-5, and IL-13 cytokines, together with marked eosinophilia (22). It is difficult to compare our findings to the existing literature since this is the first study to provide cumulative evidence about the impact of CRS on COVID-19. However, asthma is frequently found in CRS patients and is usually associated with type 2 inflammation (23). Previous studies investigated the impact of asthma on developing COVID-19 and compared affected patients’ outcomes between patients with and without asthma. For instance, a previous meta-analysis by Sunjaya et al. (24) reported that the risk of SARS-CoV-2 infection was significantly reduced among patients with asthma, with an estimated pooled prevalence of COVID-19 of 8.08% among these patients, which is slightly higher than ours. A more updated meta-analysis furtherly demonstrated that the risk of infection was significantly lower among patients with asthma than others (25). Accordingly, it can be concluded that CRS, similar to asthma, is not associated with higher rates of SARS-CoV-2 infections.

Regarding COVID-19 severe outcomes, CRS was significantly associated with increased hospitalization rates. On the other hand, mechanical ventilation and mortality rates, and length of hospital stay were comparable between the two groups. Similarly, previous meta-analyses also demonstrated the neutral effects that asthma plays among patients with COVID-19. These studies showed that the hospitalization, MV, ICU admissions, and mortality rates were similar between COVID-19 patients with and without asthma (24, 25). Studies that reported the protective effects of these respiratory diseases proposed some factors for this phenomenon. For instance, it has been suggested that the protective effect of CRS might be related to the expression of the angiotensin-converting enzyme (ACE)-2 receptor in CRS patients. This has been indicated in the analysis by Wang et al. (26), which demonstrated that ACE-2 receptor expression is significantly reduced among patients with eosinophilic CRS with nasal polyps (ECRSwNP). Therefore, although increased expression of ACE-2 receptors was not noticed in the non-eosinophilic CRSwNP type, this evidence indicates that some CRS patients might have a protective effect against COVID-19. This can partially explain the current results. However, a definite explanation could not be reached because we could not conduct a proper analysis that might explain the impact of different CRS subtypes. Moreover, even if we assumed that most patients included in the current analysis have ECRS [which usually presents with more severity and worsened outcomes (27–31)], our results would still show that patients with CRS have comparable outcomes to those without it, excluding the negative impact of CRS on COVID-19 outcomes.

Another important factor to consider is the impact of steroid administration on COVID-19 outcomes among CRS patients. Our findings indicate that the administration of steroid/immunosuppressive therapy did not significantly impact the risk of acquiring COVID-19 in CRS patients. Many concerns have arisen among these patients because of their prolonged administration of steroids, as reports showed that it might impact their immune functions and induce the severity of COVID-19 (32, 33). On the other hand, other investigations demonstrated that administering corticosteroids in patients with respiratory allergic diseases associated with type 2 inflammation is associated with the downregulation of nasal cavity ACE-2 receptors, leading to a reduced risk of SARS-CoV-2 infection (32, 34, 35). This might explain why COVID-19 cases are not high and do not suffer from more severe outcomes in the CRS compared to the control group. Similar conclusions were also reported in studies investigating the impact of asthma (34). Therefore, the administration of steroid therapy in CRS patients should not be avoided, especially to reduce the risk of sneezing and spreading the infection (36). However, it should be noted that these findings were more remarkable in patients with eosinophilia. Moreover, one included study reported that the administration of steroid/immunosuppressive therapy was associated with more severe COVID-19 in CRS patients. However, the authors demonstrated that this finding is not clinically significant and needs further confirmation (18). Although biologics might be used in CRS patients with favorable outcomes, evidence regarding its impact on SARS-CoV-2 infection and severity of outcomes in these patients is limited and needs further investigations (37). However, studies recruiting patients with atopic dermatitis showed that the drug has no effect on acquiring COVID-19 and does not induce severe outcomes (38, 39). However, using these modalities is still controversial and needs further validation (40).

A third factor to be considered is the presence of comorbidities and the administration of treatment regimens that can be protective against COVID-19. However, we found that male sex, cardiovascular morbidity, renal diseases, and hypertension were inversely associated with COVID-19, which contradicts the findings reported by the previous COVID-19 investigation (41–43). The exact reason for this contradiction is unknown. However, it might be due to the potential differences in baseline characteristics of included patients and different management approaches that might reduce the impact and intensity of these comorbidities. The current analysis could not adjust confounders due to data shortage and improper study designs. Accordingly, further future investigations with proper designs and better adjustment of baseline characteristics of their populations are urgently needed.

There are some limitations to be considered in the current analysis. First, many of the included studies did not have proper designs and sample sizes, which might not be sufficient to conduct an ideal analysis that identifies the association between CRS and COVID-19 without the impact of certain confounders, like demographics, treatment regimens, and associated morbidities. Randomized controlled trials are encouraged in this context. Moreover, limitations within included studies should also be considered. For instance, Wang et al. (13) reported that the risk of developing severe COVID-19 was not associated with CRS. However, they also mentioned that deceased patients with CRS were excluded from the study. These could have been patients with severe COVID-19. Therefore, their data might not have been adequately representative.

## 5 Conclusion

The impact of CRS on COVID-19 seems neutral. Neither CRS nor related steroid/immunosuppressive therapy increases the risk of acquiring COVID-19. Besides, although CRS increases the risk of hospitalization in COVID-19 patients, it does not induce severe outcomes, including length of hospital stay, ICU admissions, mechanical ventilation, and mortality.

## Data availability statement

The original contributions presented in the study are included in the article/**Supplementary Material**. Further inquiries can be directed to the corresponding authors.

## Author contributions

AR was responsible for the idea and the study design. All authors shared in the data extraction. AR analyzed the data and interpreted it. All authors shared in the writing of the full text and approval of final version before submission. All steps of this study were supervised by AE-Q. All authors contributed to the article and approved the submitted version.
