# Supplementary material for: The impact of chronic rhinosinusitis on COVID-19 risk and outcomes: A systematic review and meta-analysis
Source: Front Immunol. 2023 Mar 29;14:1139031. doi: 10.3389/fimmu.2023.1139031 (PMC10090854; doi:10.3389/fimmu.2023.1139031)
Supplement: Supplementary file 1 [file DataSheet_1.docx]

**Supp. Table 1**. Quality assessment of Cross-sectional studies using the modified Newcastle Ottawa scale.

| **Author** | **Year** | **Selection** | | | | **Comparability** | **Outcome** | | **Total** |
| --- | --- | --- | --- | --- | --- | --- | --- | --- | --- |
|  |  | **RepresentaTiveness of the Sample** | **Sample size** | **Non Respondents** | **Ascertainment of the Exposure** | **The Subjects in Different Outcome Groups are Comparable** | **Assessment of outcome** | **Statistical analysis** |  |
| **Akhlaghi et al.** | 2021 | * | * |  | * | * | * | * | 6 |
| **Faiq et al.** | 2022 | * | * | * | * | * | * | * | 7 |
| **Sbeih et al.** | 2022 | * | * | * | * | * | * | * | 7 |
| **Wang et al.** | 2020 | * | * | * | * | * | * | * | 7 |
| **Workman et al.** | 2021 | * | * |  | * |  | * | * | 5 |

A study can be awarded a maximum of one star for each numbered item within the Selection and Exposure categories. A maximum of two stars can be given for Comparability and ascertainment of the exposure within the selection domain. Accordingly, items of “selection” can be rated by a maximum of 5 stars, Comparability by 2 stars, and Exposure by 3 stars, with a maximum of 10 stars (High quality: 7-10, Moderate quality: 4-6, low quality: 0-3). The scale was first modified by Herzog et al. (1) for cross-sectional studies and used by various subsequent studies.

**Supp. Table 2**. Quality assessment of cohort studies using the Newcastle Ottawa scale.

| **Author** | **Year** | **Selection** | | | | **Comparability** | **Outcome** | | | **Total** |
| --- | --- | --- | --- | --- | --- | --- | --- | --- | --- | --- |
|  |  | **Representativeness of the exposed cohort** | **Selection of the non-exposed cohort** | **Ascertainment of exposure** | **Demonstration that outcome of interest was not present at the start of the study** | **Control for important or additional factors** | **Assessment of outcome** | **Was follow-up long enough for outcomes to occur** | **adequacy of follow-up of cohorts** |  |
| **Lee et al.** | 2021 | * | * | * | * | ** | * | * | * | 9 |
| **Miller et al.** | 2021 | * | * | * | * | * | * |  |  | 6 |

A study can be awarded a maximum of one star for each numbered item within the Selection and Exposure categories. A maximum of two stars can be given for Comparability.” Accordingly, items of “selection” can be rated by a maximum of 4 stars, Comparability by 2 stars, and Exposure by 3 stars, with a maximum of 9 stars (High quality: 7-9, Moderate quality: 4-6, Low quality: 0-3). The scale can be found at: https://www.ohri.ca/programs/clinical_epidemiology/oxford.asp

**References**

1. Herzog R, Álvarez-Pasquin MJ, Díaz C, Del Barrio JL, Estrada JM, Gil Á. Are healthcare workers’ intentions to vaccinate related to their knowledge, beliefs and attitudes? a systematic review. BMC Public Health. 2013;13(1):154.
